# Growth performance, organ weight, fecal scores, plasma, and ceca digesta microbial metabolites in growing pigs fed spent biomass of *Pichia kudriavzevii*

**DOI:** 10.1093/tas/txaa152

**Published:** 2020-08-11

**Authors:** Elijah G Kiarie, Conor Voth, Doug Wey, Cuilan Zhu, Lee-Anne Huber, E James Squires

**Affiliations:** Department of Animal Biosciences, University of Guelph, Guelph, ON Canada

**Keywords:** growth performance, *Pichia kudriavzevii*, pigs, plasma and digesta metabolites, organ weight

## Abstract

Growth performance, liver and spleen weight, plasma, and ceca digesta metabolites and incidences of diarrhea were investigated in growing pigs fed spent biomass of *Pichia kudriavzevii.* Ninety six barrows (~25 kg, 4 pigs/pen) were fed 1 of 4 experimental diets (*n* = 6) for 7 weeks. The diets were control, corn-, and soybean meal-based diet or control plus 2.5%, 3.75%, or 5.0% *P. kudriavzevii*. Diets were formulated to be isocaloric and iso nitrogenous. Feed intake and body weight (BW) were recorded weekly for calculation of average daily gain (ADG), average daily feed intake (ADFI), and gain to feed ratio (G:F). Fecal scores were taken 3 d/wk to assess incidence and severity of diarrhea. One pig/pen close to pen average was bled for plasma metabolites on days 7 and 49 and subsequently euthanized for spleen and liver weight, ileal and cecum digesta samples for concentration of short-chain fatty acids (SCFA). The concentration of crude protein, crude fat, and non-fiber carbohydrates in *P. kudriavzevii* biomass was 36.4%, 9.6%, and 50.8% DM, respectively. Inclusion of *P. kudriavzevii* tended (*P* = 0.06) to linearly reduce ADG from days 8 through 49 resulting in a trend (*P* = 0.06) for linear reduction in the final BW. The final BW was 79.0, 79.2, 76.8, and 75.5 kg for the 0%, 2.5%, 3.75%, and 5.0% *P. kudriavzevii*, respectively. Diets had no effect (*P* > 0.10) on ADFI, G:F, spleen, and liver weight throughout the entire experiment. On day 7, there was cubic (*P* = 0.03) decrease and quadratic (*P* = 0.02) increase in plasma concentration of creatinine and urea N, respectively. However, there were no (*P* > 0.10) diet effects on plasma metabolites on day 49. There was a tendency (*P* = 0.08) for linear increase in cecum digesta concentration of acetic acid. There were no diet effects (*P* > 0.10) on fecal score in the first 4 wk of feeding. In conclusion, feeding *P. kudriavzevii* yeast tended to depress growth and stimulate cecum fermentation at higher dose and had no detrimental effects on organ weights or plasma metabolites in growing pigs.

## INTRODUCTION

There are more than 1,000 known species of yeast but very few are commercially exploited (Kurtzman et al., 2011). The genus *Saccharomyces* has approximately 20 species that are of significant importance in alcoholic fermentation, bread-making, single cell protein and vitamin production, synthesis of recombinant proteins, and other applications (Kurtzman et al., 2011). Indeed, as the most important species in this genus, the annual global production of *Saccharomyces cerevisiae* is at a level that exceeds the combined production of all other industrial microorganisms by about two orders of magnitude (Jansen et al., 2017). Additionally, *Candida utilis* (formerly classified as *Torulopsis utilis*) commercially known as “Torula Yeast” is unique as it utilizes pentose sugars, making it very useful in processing wood pulp to paper. Another important yeast is *Kluyveromyces marxianus* or the “whey yeast” for dairy processing. However, advancement in molecular biology has opened tremendous opportunities for developing other yeast strains for diverse applications (Øverland and Skrede, 2017; Douglass et al., 2018).

*Pichia kudriavzevii* is one of most widely distributed yeast isolates, often involved in spontaneous fermentations and has been used for centuries to produce several traditional fermented cultural foods across the world (Douglass et al., 2018). Because of its exceptional acid tolerance, *P. kudriavzevii* has a growing role in biotechnology for bioethanol fermentation (Radecka et al., 2015; Mukherjee et al., 2017) and synthesis of high-value platform chemicals such as succinic acid (Xiao et al., 2014; Douglass et al., 2018; Rush, 2018). An inactivated biomass derived from *P. kudriavzevii* strain that has been developed to produce succinic acid using corn syrup as carbon source was characterized to be rich in crude protein, fat, and carbohydrates (Rush, 2018). The objective of the present study was to assess effects of 2.5%, 3.75%, and 5.0% inclusion of *P. kudriavzevii* biomass in complete diet for growing pigs on growth performance, organs weight, plasma, ceca digesta metabolites, and fecal consistency score.

## MATERIALS AND METHODS

Animal care and use protocol (#3838) was approved by the University of Guelph Animal Care and Use Committee and pigs were cared for in accordance with the Canadian Council on Animal Care guidelines (CCAC, 2009). A research exemption (#1710043) was granted by Canadian Food Inspection Agency pursuant to Feed Acts and Regulations for feeding unregistered spent *P. kudriavzevii* biomass to pigs.

### Spent Yeast Biomass and Feed Preparations

Heat-inactivated *P. kudriavzevii* biomass was sourced from a bio refinery plant (LCY Biosciences, formerly BioAmber Sarnia Inc., Sarnia, ON, Canada). The chemical characterization of the *P. kudriavzevii* sample is shown in Table 1. Two diets, a corn- and soybean meal-based diet without (0% *P. kudriavzevii*) or with 5% DM *P. kudriavzevii* were formulated to meet or exceed the estimated nutrient recommendations (NRC, 2012) for growing pigs (Table 2). Two additional diets were made by proportional mixing of control and 5% *P. kudriavzevii* diet to create 2.5% and 3.75% DM *P. kudriavzevii* diets. The metabolizable energy (ME) and standardized ileal digestibility (SID) of amino acids (AA) for *P. kudriavzevii* were from whole yeast values for pigs from AMINODat 5.0 database (Evonik, 2016). The *P. kudriavzevii* biomass was received at the research station as liquid (75% moisture) and as such, the 5% *P. kudriavzevii* diet was manufactured with equivalent (20% *P. kudriavzevii*) on fed basis. Briefly, all the other components of 5%*P. kudriavzevii* diet (Table 2) were mixed as one batch and the *P. kudriavzevii* was added for each weekly allotment of feed and stored in a cooler; only the projected portion to be eaten within a few days was placed in the feeders in the pen. Representative feed samples were collected at the time of mixing and regularly at the pen for dry matter determination on a weekly basis.

**Table 1.** Chemical composition (dry matter basis) of the spent *P. kudriavzevii* biomass

Item	Amount
Moisture, %	75.0
Crude protein, %	36.4
Crude fiber, %	1.07
Crude fat, %	9.62
Crude ash, %	3.08
Calcium, %	0.10
Phosphorous, %	0.60
Non-fiber carbohydrates, %	50.75
Leucine, %	3.43
Isoleucine, %	2.52
Lysine, %	3.07
Methionine, %	0.80
Cysteine, %	0.44
Threonine, %	2.24
Valine, %	2.40

**Table 2.** Composition of experimental diets^*a*^, as fed basis

Ingredient, %	Yeast inclusion, %	
	0.0	5.0
Corn	74.8	73.6
Soybean meal	20.7	17.1
Yeast biomass	–	5.00
Soybean oil	1.39	1.28
Monocalcium phosphate	0.98	0.91
Limestone	0.91	0.95
Vitamin trace mineral premix^*b*^	0.50	0.50
L-Lysine HCL	0.35	0.31
Salt	0.22	0.22
L-Threonine	0.09	0.07
DL-Methionine	0.07	0.06
L-Tryptophan	0.02	0.02
Calculated provisions		
Metabolizable energy, kcal/kg	3,300	3,300
Crude protein, %	16.0	16.0
SID Lysine, %	0.98	0.98
SID Methionine, %	0.31	0.32
SID Methionine + Cysteine, %	0.55	0.55
SID Tryptophan, %	0.17	0.17
SID Threonine, %	0.59	0.59
Digestible P, %	0.31	0.31
Calcium, %	0.66	0.66
Sodium, %	0.10	0.10
Chloride, %	0.22	0.22

^*a*^Diets 0% and 5% yeast were blended proportionally to create 2.5% and 3.75% yeast diets.

^*b*^Provided per kg of premix: vitamin A, 2,000,000 IU as retinyl acetate; vitamin D_3_, 200,000 IU as cholecalciferol; vitamin E, 8,000 IU as dl-α-tocopherol acetate; vitamin K, 500 mg as menadione; pantothenic acid, 3,000 mg; riboflavin, 1,000 mg; choline, 100,000 mg; folic acid, 400 mg; niacin, 5,000 mg; thiamine, 300 mg; pyridoxine, 300 mg; vitamin B_12_, 5,000 mcg; biotin, 40,000 mcg; Cu, 3,000 mg from CuSO_4_×5H_2_O; Fe, 20,000 mg from FeSO_4_; Mn, 4,000 mg from MnSO_4_; Zn, 21,000 mg from ZnO; Se, 60 mg from Na_2_SeO_3_; and I, 100 mg from KI (DSM Nutritional Products Canada Inc., Ayr, ON, Canada).

### Animals, Housing, and Experimental Procedures

A total of 96 growing barrows crossbred (Yorkshire × Landrace ♀ × Duroc ♂; approximately 25-kg body weight) were procured from University of Guelph Arkell swine research station (Guelph, ON). The pigs were allocated to pens (4 pigs per pen) based on BW in two environmentally controlled rooms at Arkell swine research station. Each room had 12 pens, each measuring (76″ × 168″) and equipped with a feeder, a nipple type drinker, plastic-covered expanded metal floors, and a wall partitioning between pens that allowed visual contact with pigs in adjacent pens. Room temperature was set at 22 °C. The four dietary treatments were allocated in a completely randomized design to give six replicate pens per diet. Feed intake and body weight were determined weekly for calculation of average daily gain, feed intake, and gain:feed. The occurrence and severity of diarrhea was monitored and assessed on a pen basis 3 d per week using a fecal consistency scoring system (1 = normal; 2 = soft feces; 3 = mild diarrhea; 4 = severe diarrhea) between days 8 and 14 (Kiarie et al., 2011). One pig per pen close to the pen average BW was bled and killed on day 7 (1 wk after feeding) and on day 49 (last day of feeding). Blood samples (10 mL) were collected from orbital sinus bleeding technique (Dove and Alworth, 2015) using a Monoject Standard Hypodermic needle 16 G × 1″ (Covidien; Mansfield, MA) into vacutainer tubes coated with lithium heparin (Becton Dickinson & Co, Franklin Lakes, NJ). The samples were immediately centrifuged at 2,000 × *g* for 10 min at 4 °C to recover plasma, which was immediately stored at −20 °C until used for analyses. For sacrifice, the pig was sedated with a premix of 0.2 mL kg-1 BW [1 mL contained: Ketamine (50 mg), butorphanol (1 mg), and xylazine (10 mg)] via intramuscular injection followed by intravenous injection of Pentobarbital (Euthansol) at 68 mg kg^−1^ BW. The spleen and liver were removed, blotted dry with paper towels, and weighed. The ileal and cecum digesta samples from pigs killed on day 7 were placed in plastic bags and immediately frozen for short-chain fatty acids (SCFA) analyses. All pigs fed yeast were killed at the end of the trial and were composted along with the leftover feed and liquid yeast.

### Laboratory Analyses

Weekly feed samples were analyzed for dry matter according to standard procedures method 930.15 (AOAC, 2005). The concentration of SCFA (lactic, formic, acetic, propionic, isobutyric, and n-butyric) in the ceca digesta was assayed according to Leung et al. (2018). Briefly, the digesta was thawed and approximately 0.1 g was resuspended with 1 mL 0.005N H_2_SO_4_ (1:10, wt/vol) in a microcentrifuge tube. The tube was vortexed vigorously until sample was completely dissolved, centrifuged at 11,000 × *g* for 15 min, 400-µL supernatant transferred into a high-performance liquid chromatography (HPLC) vial, and 400 µL of 0.005N H_2_SO_4_ buffer added. The resulting digesta fluid was then assayed for SCFA using HPLC (Hewlett Packard 1100, Germany) with Rezex ROA-Organic Acid LC column, 300 × 7.8 mm from Phenomenex and Refractive Index detector at 40 °C (Agilent 1260 Infinity RID from Agilent Technologies, Germany). Twenty microliters of the resulting sample was injected into the column, with a column temperature of 60 °C and mobile phase of 0.005N H_2_SO_4_ buffer at 0.5mL/min isocratic for 35 min. The detector was heated to 40 °C. The plasma urea nitrogen and creatinine were analyzed by photometrics using a Roche Cobas 6000 c501 biochemistry analyzer (Roche Diagnostics USA, Indianapolis, IN) at the Animal Health Laboratory (University of Guelph, Guelph, ON).

### Calculation and Statistical Analyses

For calculation of ADFI and G:F, the feed intake data for 2.5%, 3.75%, and 5% *P. kudriavzevii* diets were standardized to the DM content of the control diet using the weekly feed DM determinations. Data were analyzed using the PROC MIXED procedures of SAS (v. 9.4 SAS Institute Inc., Cary, NC) with pen as the experimental unit. The model had diet as fixed effect and block (room) as the random effect. Coefficients for linear and quadratic effects of *P. kudriavzevii* inclusion were generated using IML procedures of SAS. An α level of *P* ≤ 0.05 was used as the criteria for assessing for statistical significance and trends (0.05 < *P* ≤ 0.10) were discussed.

## RESULTS AND DISCUSSION

There was no mortality or medication given to any pigs throughout the trial and pigs readily consumed feed. The average feed DM content was 88.3%, 83.7%, 80.6%, and 77.7% for 0%, 2.5%, 3.75%, and 5.0% *P. kudriavzevii*, respectively (Table 3). Although there were no diet effects (*P* > 0.10) on ADG in days 0 to 7, a tendency (*P* = 0.06) for a linear decrease in ADG was observed for days 8 to 49 (Table 4). Subsequently, there was a tendency for a linear decrease in final BW. The final BW was 79.0, 79.2, 76.8, and 75.5 kg for 0.0%, 2.5%, 3.75%, and 5.0% *P. kudriavzevii*, respectively. There were no diet effects (*P* > 0.10) on ADFI and G:F throughout the experiment. However, numerically, pigs fed 3.75% and 5.0% *P. kudriavzevii* consumed 4.5% and 5.0% less feed than pigs fed 0% *P. kudriavzevii* days 8 to 49, suggesting that the trends for decreased BW at higher doses of *P. kudriavzevii* were partly due to feed intake depression. Although not measured in the present study, the concentration of nucleic acids has been linked to reduced feed palatability (Rumsey et al., 1991). The total nucleic acids concentration in whole yeast has been reported to be between 6% and 12% dry cell weight (Waldron and Lacroute, 1975; Běhalová et al., 1991). However, pigs fed different level of yeast products showed variable growth performance responses. For example, feeding nursery pigs 3% brewers’ yeast depressed growth due to reduced feed intake (White et al., 2002). Growth and feed intake was estimated to start declining at 1.91% in nursery pigs fed 0.0, 1.0, 2.0, and 3.0 *Saccharomyces cerevisae* yeast extract (Pereira et al., 2012). Other studies have indicated contrary results. For example, a series of experiments showed that 4% or 5% dried brewers’ yeast had no effects on growth performance in nursing and nursery pigs (LeMieux et al., 2010). *Candida utilis* dried biomass fed at 10%, 20%, and 30% had no effects on growth and feed intake in nursery pigs (Cruz et al., 2019).

**Table 3.** Weekly dry matter (%) content in mixed feed

Week	Yeast inclusion, %			
	0.0	2.5	3.75	5.0
1	88.8	85.3	83.4	81.8
2	88.2	84.4	80.8	78.6
3	88.5	83.1	80.9	77.2
4	87.8	83.1	78.8	77.1
5	87.8	82.8	80.1	75.7
6	88.3	83.5	79.5	76.1
7	88.4	83.7	81.1	77.8
Average	88.3	83.7	80.6	77.7

**Table 4.** Effects of spent *P. kudriavzevii* biomass on growth performance, organ weights, and plasma metabolites in growing finishing pigs

Item	Yeast inclusion, %				SEM	Response curve		
	0	2.5	3.75	5.0		Linear	Quadratic	Cubic
Growth performance								
Days 0–7^*a*^								
Initial BW, kg	24.7	25.2	25.0	25.4	1.158	–	–	–
Final BW, kg	31.0	31.0	31.1	31.6	1.600	0.770	0.910	0.981
ADG, g/d	896	830	864	892	79.94	0.897	0.619	0.686
ADFI^*b*^, g/d	1,401	1,430	1,265	1,591	97.26	0.207	0.126	0.237
G: F, g/g	0.639	0.574	0.692	0.576	0.050	0.579	0.448	0.100
Days 8 to 49^*c*^								
Initial BW, kg^*d*^	31.1	31.1	31.1	31.5	1.581	–	–	–
Final BW, kg	79.0	79.2	76.8	75.5	1.384	0.055	0.962	0.453
ADG, g/d	1,138	1,143	1,087	1,057	33.02	0.056	0.962	0.453
ADFI, g/d	2,659	2,683	2,539	2,525	108.1	0.291	0.886	0.506
G: F, g/g	0.428	0.434	0.439	0.422	0.021	0.812	0.613	0.924
Days 0 to 49								
ADG, g/d	1,102	1,097	1,054	1,034	34.05	0.136	0.869	0.619
ADFI, g/d	2,510	2,426	2,288	2,326	121.1	0.274	0.437	0.720
G: F, g/g	0.440	0.458	0.464	0.452	0.020	0.754	0.413	0.970
Liver weight, g/kg BW								
Day 7	26.2	25.8	26.8	25.4	0.889	0.572	0.502	0.432
Day 49	17.9	19.0	18.6	18.6	0.671	0.644	0.460	0.431
Spleen weight, g/kg BW								
Day 7	1.58	1.92	1.85	1.93	0.135	0.154	0.317	0.317
Day 49	1.60	1.64	1.55	1.63	0.120	0.967	0.803	0.584
Plasma metabolites, mmol/L								
Day 7								
Creatinine	63.3	53.3	59.7	56.8	2.57	0.300	0.262	0.026
Urea N	1.35	1.62	2.07	1.33	0.22	0.921	0.020	0.356
Day 49								
Creatinine	99.2	93.2	95	96.7	4.339	0.867	0.415	0.580
Urea N	4.42	4.83	4.08	5.52	0.496	0.217	0.341	0.234

^*a*^4 pigs/pen (*n* = 6).

^*b*^Feed intake data for 2.5%, 3.75%, and 5% *P. kudriavzevii* diets were standardized to the DM content of the control diet using the weekly feed DM determinations (Table 3).

^*c*^3 pigs/pen (*n* = 6).

^*d*^Used as covariate for statistical analyses of days 8 to 49 growth data.

Yeasts are rich in endogenous nucleases that can degrade nucleic acids into nucleotides through autolysis (Běhalová et al., 1991; Chaffin et al., 1998). Exogenous purine nucleosides and nucleotides were demonstrated to stimulate DNA synthesis in cultured renal epithelia cells (Toback et al., 1990). The concentration of nucleotides was not determined in the current study; however, spleen and liver were assessed to give metabolic indication of feeding spent *P. kudriavzevii* biomass. There were no diet effects (*P* > 0.10) on liver and spleen weight (Table 4). This contrasted with previous research that indicated yeast derivatives induced organ hypertrophy in pigs and poultry (Kiarie et al., 2011; Waititu et al., 2017; Leung et al., 2019). Plasma metabolites also serve as markers for animal health status (physiological, nutritional, pathological changes). Dietary nucleic acids can result in elevated plasma urea N indicative of toxicological effects as well as disturbances in protein, fat, carbohydrate, and uracil metabolism (de Oliveira and Burini, 2012). Diets affected (*P* ≤ 0.03) plasma creatinine and urea N in a non-linear fashion on day 7 only (Table 4). For creatinine, pigs fed 2.5% *P. kudriavzevii* showed lower concentration than control whilst pigs fed 3.75% *P. kudriavzevii* had higher concentration of urea N than control and 5.0% *P. kudriavzevii* pigs. However, plasma creatinine and urea N concentrations in the current study were within the physiological range for nursery and growing pigs [0.70–9.0 mmol PUN/L, 26–165 mmol creatinine/L (Friendship et al., 1984; Perri et al., 2017)].

Yeast cell components have been implicated in modulating gut ecology through their prebiotic and immunomodulatory properties, which in turn can benefit gut health and improve growth performance (van der Aa Kühle et al., 2005; Kiarie et al., 2011; Kiarie et al., 2012; Kiarie et al., 2019). There was no diet effect (*P* > 0.10) on the concentration of lactic and acetic acids in the ileal digesta (Table 5). At the ceca level, there was a tendency (*P* = 0.08) for linear increase in concentration of acetic acid (Table 5). The concentration of other organic acids was not affected (*P* > 0.10). Results of the current study showing minimal effects of yeast biomass on digesta concentrations of fermentation products are generally in agreement with the findings of White et al. (2002). Fecal consistency score was evaluated as a gross indicator of gastrointestinal health (Table 5). There were no diet effects (*P* > 0.10) on fecal score in the first 4 wk of feeding. However, a linear (*P* = 0.01) increase in fecal score was observed on week 7. It seemed that pigs fed 2.5% *P. kudriavzevii* had lower fecal score than pigs fed 5.0% *P. kudriavzevii*. These observations were in contrast with a study in which pigs fed 10%, 20%, and 30% of *Candida utilis* biomass had lower fecal score and higher fecal dry matter than the control (0%) (Cruz et al., 2019). As heterotrophic organisms, energy and carbon metabolism are intimately interconnected giving yeast cells ability to produce a wide variety of metabolites depending on the composition of the fermentation media and the fermentation conditions (Hatoum et al., 2012). In this context, it is plausible that yeast biomass and yeast derivatives used in different studies may differ in terms of metabolites that may have nutritional and health effects in animals.

**Table 5.** Effects of spent *P. kudriavzevii* biomass on concentration of short-chain fatty acids (SCFA) in ileal and cecal digesta and fecal consistency score

Parameter	Yeast inclusion, %				SEM	Response curve		
	0	2.5	3.75	5.0		Linear	Quadratic	Cubic
Digesta SCFA, µmol/mL								
Ileal								
Lactic acid	22.8	27	41	43.3	12.508	0.213	0.704	0.682
Acetic acid	6.94	8.81	5.7	6.91	1.846	0.751	0.921	0.263
Cecum								
Lactic acid	1.45	4.79	8.02	6.42	4.47	0.446	0.468	0.875
Acetic acid	66.5	71.7	70.8	76.5	3.642	0.083	0.919	0.506
Propionic	38.7	41.8	42.1	47.4	4.833	0.216	0.949	0.785
Isobutyric	3.83	3.18	2.36	3.44	0.977	0.802	0.318	0.758
Butyric	17.9	15.0	16.6	15.2	1.928	0.460	0.748	0.382
Fecal consistency score^*a*^								
Week 1	2.75	2.20	2.52	2.77	0.196	0.509	0.104	0.163
Week 2	2.77	2.33	2.58	2.73	0.165	0.692	0.154	0.181
Week 3	2.65	2.42	2.47	2.87	0.173	0.243	0.121	0.771
Week 4	2.88	2.42	2.58	2.80	0.139	0.835	0.038	0.182
Week 5	2.85	2.42	2.77	2.93	0.149	0.279	0.142	0.080
Week 6	2.75	2.33	2.83	3.00	0.172	0.094	0.304	0.062
Week 7	2.55	2.35	2.77	2.92	0.127	0.013	0.574	0.074

^*a*^Fecal score system; 1, normal; 2, soft feces; 3, mild diarrhea; 4, severe diarrhea (Kiarie et al., 2011).

Yeasts have long been cultivated as rich source of protein, minerals, vitamins (particularly B vitamins), and other nutrients for humans and animals. Production of single cell protein from yeast has been suggested to have tremendous advantages relative to plant, animal, and other microbial sources of protein because of their rapid growth rate on a wide variety of substrates, including industrial and agricultural waste (Ugalde and Castrillo, 2002; Øverland and Skrede, 2017). The current data suggested that *P. kudriavzevii* yeast biomass had tendency to decrease growth at more than 3.75% inclusion but had no detrimental effects on organs and metabolism in growing pigs when fed up to 5.0%. The product can thus be incorporated in swine feeding programs.
